# Role of Aryl Hydrocarbon Receptor Activation in Inflammatory Chronic Skin Diseases

**DOI:** 10.3390/cells10123559

**Published:** 2021-12-16

**Authors:** Maddalena Napolitano, Gabriella Fabbrocini, Fabrizio Martora, Vincenzo Picone, Paola Morelli, Cataldo Patruno

**Affiliations:** 1 Department of Medicine and Health Sciences Vincenzo Tiberio, University of Molise, 86100 Campobasso, Italy; 2 Section of Dermatology, Department of Clinical Medicine and Surgery, University of Naples Federico II, 80131 Naples, Italy; gafabbro@unina.it (G.F.); fabriziomartora92@libero.it (F.M.); vince.picone95@gmail.com (V.P.); 3 Department of Health Sciences, University Magna Graecia of Catanzaro, 88100 Catanzaro, Italy; paola.morelli@unicz.it (P.M.); cataldopatruno@libero.it (C.P.)

**Keywords:** aryl hydrocarbon receptor, atopic dermatitis, psoriasis, acne, hidradenitis suppurativa

## Abstract

Aryl Hydrocarbon Receptor (AhR) is an evolutionary transcription factor which acts as a crucial sensor of different exogenous and endogenous molecules Recent data indicate that AhR is implicated in several physiological processes such as cell physiology, host defense, proliferation and differentiation of immune cells, and detoxification. Moreover, AhR involvement has been reported in the development and maintenance of several pathological conditions. In recent years, an increasing number of studies have accumulated highlighting the regulatory role of AhR in the physiology of the skin. However, there is evidence of both beneficial and harmful effects of AHR signaling. At present, most of the evidence concerns inflammatory skin diseases, in particular atopic dermatitis, psoriasis, acne, and hidradenitis suppurativa. This review exam-ines the role of AhR in skin homeostasis and the therapeutic implication of its pharmacological modulation in these cutaneous inflammatory diseases.

## 1. Introduction

Aryl Hydrocarbon Receptor (AhR), a ligand-dependent transcription factor, is known to mediate the biochemical and toxic effects of xenobiotics, environmental stresses, endogenous ligands, microbial-derived products, and physiological compounds such as tryptophan derivatives [1,2,3,4,5]. Recent data indicate that AhR is implicated in several physiological processes such as xenobiotic metabolism, cell cycle regulation, reproduction, development, and immune response, by playing a pivotal role in signaling networks [5,6,7]. Moreover, AhR involvement has been described in the pathogenesis of different diseases [7].

AhR signaling appears to play an important role in maintaining skin homeostasis by regulating metabolism of environmental toxins, oxidative stress, photoinduced response, keratinocytes differentiation, epidermal barrier function, melanogenesis, and skin immune network [8,9,10]. Several studies showed that the positive or negative biological consequences of AhR activation in the skin are highly dependent on the presence or absence of a pathological condition, the specific ligand triggering AhR activation or inhibition, and other contributing factors [11,12]. In healthy skin, AhR is constitutively active, and canonical and non-canonical-mediated signaling processes are tightly balanced [13]. In xenobiotic AhR ligand–exposed skin, canonical AhR signaling may become dominant and lead to a set of adverse effects such as increased expression of reactive intermediates, aging, or development of skin cancer [13]. Conversely, in chronically inflamed skin disease, such as atopic dermatitis (AD) and psoriasis, high levels of non-canonical AhR-partner molecules are expressed [13] (Table 1).

General observations regarding AhR function in the skin are also complicated by the responses of various cell types found in the skin, including keratinocytes, dermal fibroblasts, Langerhans cells (LCs), melanocytes, sebocytes, and immune skin cells (mast cells, CD8+ T cells, and dendritic cells (DCs)), all of which have been shown to express AhR at different levels [8]. AhR expression correlates very well with the differentiation status of the skin cells. Proliferating keratinocytes show low nuclear AhR levels and are greatly unresponsive to ligand activation, whereas differentiated cells have high cytoplasmic receptor levels [14].

We performed a narrative review of the international literature regarding the role of AhR and therapeutic implication of its pharmacological modulation in some skin diseases.

## 2. Aryl Hydrocarbon Receptor Signaling

To exert its role at transcriptional level, AhR forms a heterodimer with its Class II partner Aryl Hydrocarbon Receptor Nucleus Translocator (ARNT), thus recognizing a specific Xenobiotic Response Element (XRE, or DRE for Dioxin Response Element) in the promoter of downstream genes [15]. Both AhR and ARNT contain a transactivation domain (TAD) in their C-terminal region, mediating the transcription initiation by recruiting transcription factors and co-regulators to the transcriptional site [16,17].

**Figure 1:**
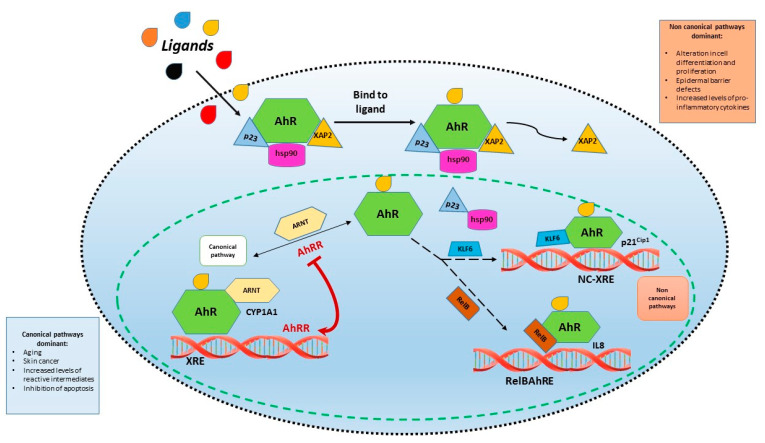
Canonical and non-canonical signaling pathways activated by AhR. In canonical pathways upon ligand-binding by either exogenous or endogenous agonists, AhR undergoes conformational changes leading to the dissociation of p23 and XAP2 with consequent translocation in the nucleus and formation of a AhR:ARNT heterodimer. The AhR:ARNT complex binds to upstream regulatory regions of its target genes which contain canonical aryl hydrocarbon XRE elements. In non-canonical pathway ligand-AhR complex can interact directly with sites distinct from the consensus XRE.

Canonical and non-canonical signaling pathways activated by AhR have been identified (Figure 1). Canonical AhR is described in detail in several studies [18,19]. In inactive state, AhR is located in the cytoplasm in a protein complex including a dimer of 90-kDa heat shock protein (Hsp90), co-chaperones p23, and the human hepatitis B virus X-associated protein (XAP2) [20]. The chaperon complex maintains AhR in an inactive and stable conformation with a high-binding affinity for ligand and retains the receptor in the cytoplasm [4]. Upon ligand-binding by either exogenous or endogenous agonists, AhR undergoes conformational changes leading to the dissociation of p23 and XAP2, the unmasking of Nuclear Localization Signal (NLS), and the consequent translocation in the nucleus through the interaction with importin β [15]. The ligand-dependent nuclear import of AhR is negatively regulated by phosphorylation for Ser-12 or Ser-36 at the two phospho-kinase C (PKC) sites adjacent to the NLS of AhR, suggesting a two-step mechanism in the ligand-dependent nuclear translocation of AhR involving firstly a phosphorylation event, then the binding to importin β for the nuclear shuttling of the protein [21,22]. Upon nuclear translocation, Hsp90 is released with the formation of a AhR:ARNT heterodimer [15]. Then, the AhR:ARNT complex binds to upstream regulatory regions of its target genes which contain canonical aryl hydrocarbon XRE elements. The complex with DNA then recruits coactivators, which alter the chromatin structure into a more accessible configuration through histone acetyltransferase and histone methyltransferase activities. In the canonical pathway, the AhR–ARNT–XRE interactions regulate the expression of genes involved both in phase I and phase II xenobiotic-metabolizing enzymes (e.g., cytochromes P450 (CYP) 1A1, CYP1A2, CYP1B1 and UDP glucuronosyltransferase 1 family polypeptide A6), associated with adaptive or toxic responses to exogenous agonists [23,24].

The AhR non-canonical pathway occurs through alternative binding of nuclear AhR independent of ARNT and can result in expression of genes needed to maintain homeostasis [24]. Among genes recognized as AhR targets, many do not contain a consensus XRE and ligand-AhR complex can interact directly with sites distinct from the consensus XRE, such as unliganded estrogen receptor or retinoblastoma protein (RB) [24,25,26]. Indeed, some genes regulated by AhR have sequences known as non-consensus XRE (NC-XRE) containing a repeated tetranucleotide motif; thus, the interaction with ARNT is not necessary [24,27]. KLF6 is a transcriptional factor involved in several cellular processes, such as proliferation, differentiation and apoptosis, and alterations in its expression are associated with various types of malignancies [28]. NF-κβ is another example of a protein that interacts directly with AhR in the absence of ARNT AhR activity is influenced by several endogenous and exogenous ligands [29]. Endogenous ligands are metabolites derived from tryptophan catabolism, since AhR regulates the expression of enzymes of the metabolic pathway converting tryptophan in kynurenine [30]. Endogenous ligands of AhR may also derive from photo-oxidation of tryptophan, such as 6-formylindolo(3,2-b) carbazole (FICZ) [31]. Instead, diet is a source of exogenous AhR ligands; in particular, tryptophan-derived flavonoids or indoles contained in some vegetables such as Brassicaceae [29]. Indoles in the stomach are condensed in indolic agonists of AhR, while flavonoids have both agonists and antagonist activity [29]. Other exogenous AhR ligands are of microbial origin, namely from skin and gut microbiota. These ligands are mainly tryptophan metabolites acting on AhR [29]. Finally, many environmental substances may influence AhR function, including polycyclic aromatic hydrocarbons and halogenated aromatic hydrocarbons (dioxins) [29]. These substances can induce alteration of differentiation and proliferation of keratinocytes [29], as well as may induce reactive oxygen species with subsequent cellular oxidative damage [29,30,31,32].

## 3. The Role of AhR in Skin Physiology 

AHR influences skin physiology through its ability to mediate UVB stress response and antiapoptotic signaling in response to UV [33,34]. In keratinocytes, the AhR is activated in response to UVB, resulting in the up-regulation of CYP1A1 and CYP1B1 expression, and the activation of epidermal growth factor receptor (EGFR) signaling [33,35]. The explanation is that photoproducts are formed endogenously upon exposure to UVB and act as agonists for the AhR [36]. For example, some metabolites of tryptophan, an essential amino-acid that acts as the strongest natural near-UV-absorbing chromophore, represent a group of ligands for AhR involved in the induction and progression of skin cancer [37]. Additionally, the endogenous AhR ligand (FICZ) was found to be produced in human keratinocytes after exposure to UVB [36,38]. However, the effect of AhR in the presence of UVB seems to be twofold. By sensing UVB, if on the one hand AhR seems to contribute to the UV stress response system which orchestrates adaptive changes [39], on the other AhR is involved in the induction of regulatory T cells (Tregs) and in the maintenance of their suppressive activity [40]. The first activity appears to be dependent on the activation of the AhR in dendritic cells, the latter in the Tregs themselves [40]. So, AhR can be added to the list of molecular targets that the UV utilizes for exerting immunosuppression.

Another factor by which the AhR contribute to skin homeostasis is through activation by ligands of skin microflora. FICZ was detected in skin scale from patients suffering inflammatory skin diseases associated with the yeast genus *Malassezia*, a commensal skin microorganism that can become pathogenic [41]. Other high affinity AhR agonists were identified in these patient-derived extracts, including indirubin, Indolo [3,2-b] Carbazole (ICZ), tryptanthrin, malassezin, and pityriacitrin [41].

Notably, AhR:ARNT signaling has been reported to be pivotal in regulation of skin barrier structure and function [42,43,44,45,46,47,48]. In particular, the activation of the axis by environmental ligand such as dioxins, accelerates terminal epidermal differentiation, upregulating the production of aberrant skin barrier-forming proteins in vivo and in vitro [42,43]. On the other hand, both AhR and ARNT-deficient mice showed severe abnormalities in keratinization and skin barrier function [44,45]. Mechanisms by which AhR signaling enhances skin barrier function are not fully understood. However, it has been proven that AHR:ARNT initiates the expression of OVO-like 1 (OVOL1) transcription factor which subsequently improves the expression of filaggrin (FLG), hornerin (HNRN), and loricrin (LOR) proteins specific to fully differentiated keratinocytes (KCs) and corneocytes [46,47]. Finally, in skin cells the AhR appears to modulate the expression of genes such as metallo-proteinases essential for cell motility during skin development and renewal [48].

## 4. Methods

The authors followed criteria established in the Preferred Reporting Items for Systematic Reviews and Meta-Analyses (PRISMA) guidelines for this review [49]. A search of the Pubmed, Embase, and Cochrane Skin databases and that of clinicaltrials.gov was performed (until 1 October 2021). The search terms were “aryl hydrocarbon receptor”, “AhR”, “dioxin-receptor”, “atopic dermatitis”, “psoriasis”, “acne”, “hidradenitis suppurativa”. Only English-language publications were selected. Then, a revision of the abstracts and texts of the articles was made independently by each author. As a result, a total of 93 studies were selected for the evaluation in this review.

## 5. Atopic Dermatitis

AD is a chronic inflammatory T helper (Th)2 mediated skin disease clinically characterized by eczema and itch. AD is usually localized in the flexures of the limbs, face, and neck [50]. The AD rates have increased by 2- to 3-fold during the past decades in industrialized countries [51]. The latest estimates are that the prevalence of AD is about 15–20% in children and 1–3% in adults [51]. Multiple factors contribute to the AD pathogenesis, including skin barrier dysfunction, microbial dysbiosis, and immune dysregulation [52]. Interactions and crosstalk between these factors can reinforce and amplify atopic skin disease [52]. Two pathogenetic models have been proposed to explain the pathogenesis of AD: I) inside out model in which the abnormal epidermal phenotype in lesional AD skin is initiated by increased expression of cytokines that induces the epidermal abnormalities; II) an outside-in one in which AD is a disease induced by a genetic epidermal barrier defect that may trigger abnormal keratinocyte hyperplasia and secondary immune activation [53,54,55].

In some atopic subjects, loss of function mutations of FLG have been observed [56]. FLG is a protein critical for epidermal differentiation and stratum corneum function [56,57]. FLG loss-of-function mutations may influence the physical skin barrier, resulting in antigen penetration of the epidermis lower layers, activation of the immune response, and a deficit in water homeostasis [58]. It has been reported that in patients with FLG mutations AD is more persistent over time and more severe, more easily associated with allergic sensitization, and with a more important deficit of the natural moisturizing factors [59]. In addition to FLG, other components of the skin barrier have been implicated in AD, including tight junction proteins such as loricrin (LOR), involucrin (IVL) or claudin-1 [60]. All these proteins are encoded by genes in the epidermal differentiation complex (EDC) located on chromosome 1q21.3 [59]. Several studies have demonstrated that the activation of AhR is a crucial key of FLG expression in KCs [61,62,63]. Ligation of AhR to several endogenous and exogenous ligands induces its cytoplasmic-to-nuclear translocation and the expression of genes encoding FLG, LOR, IVL, and other barrier-related proteins in the EDC loci [5,42,43,64,65,66,67]. In addition, the activation of AhR up-regulates the gene and protein expression of OVOL1 transcription factor [63]. OVOL1 has been shown to be a transcriptional factor important for the expression of epidermal differentiation complex genes, including *FLG*, *IVL*, and *LOR* [46,62,68]. It was likely to be inhibited in AD skin, leading to reduced FLG expression [62,68]. The AhR activation by agonist 6-formylindolo[3,2-b]carbazole (FICZ), a tryptophan derivative, induces upregulation and nuclear translocation of OVOL1 resulting in increased FLG expression [46].

Furthermore, AD has traditionally been considered a paradigmatic type 2 immunity (T2)-driven disease [69]. In particular, interleukin-4 (IL-4) andIL-13 are produced at elevated levels in the lesional and non-lesional atopic skin and are key regulators of many of the hallmark features of AD, including epidermal hyperplasia, skin barrier dysfunction, and production of eosinophil and chemokines [69]. The central role of IL-4, IL-13, and their associated receptors in AD is best exemplified by the ongoing pursuit to pharmacologically target these cytokines and/or their signaling components in AD [69]. Recently, some studies have revealed that the Th1, Th2, Th22, and Th17 cells are also involved in the pathogenesis of AD [70]. It has been demonstrated that Th22 and Th17 immune responses contribute to chronic skin lesions of AD, especially in pediatric, intrinsic, and Asian patients [70]. Increased IL-17E levels have been found in the epidermis of AD patients and they seem to inhibit the FLG synthesis in the keratinocytes [70].

AhR activation is associated with significant interference with Th2 cytokines IL-4 and IL-13 [63]. IL-4 and IL-13 have partly shared receptor systems [69]. The binding of L-4 and IL-13 to their heterodimeric receptors activates Janus Kinase (JAK)1, JAK2, JAK3, and tyrosine kinase (TYK)2, and induces the activation (phosphorylation) of signal transducer and activator of transcription (STAT)6 [64,69]. The IL-13/IL-4-JAK-STAT6 axis inhibits both the AhR-mediated transcription of FLG, LOR, and IVL, and the cytoplasmic-to-nuclear translocation of OVOL1 reducing the expression of FLG and LOR [64,69]. AhR can also influence the itch of AD. Indeed, the gene ARNT encodes the neurotrophic factor artemin responsible for epidermal hyperinnervation and pruritus; this gene and is keratinocyte-specific and is targeted by AhR [69]. AhR activation and ARTN expression were positively correlated in the AD skin of patients, and they are associated with alloknesis, epidermal hyper-innervation, and inflammation [71]. However, how AhR regulates immune responses in sensitization phase of AD remained elusive [72]. Some authors showed that benzopyrene, a major polyaromatic hydrocarbon in smoke fume, mobilizes Langerhans cells and polarizes Th2/17 responses through the AhR, in atopic patients with a regulatory mechanism [72]. Therefore, if on the one hand the AhR stimulation induced an increased expression of proteins of the skin barrier, on the other hand the increased expression of ARTN induced by AhR seems to be involved in the development of itchy symptoms.

So, although the pathogenic role of AhR in AD is not clear, recent clinical trials have reported the efficacy of topical AhR agonist tapinarof in AD [73,74,75,76,77,78]. Tapinarof (DMVT-505; previously known as WBI-1001 and GSK2894512) is a naturally derived hydroxylated stilbene produced by bacterial symbionts of entomopathogenic nematodes with a high affinity for AhR [73,74,75,76]. Tapinarof cream displayed significant efficacy in both patients with AD or psoriasis, although its mechanism was not fully understood [79]. It activates the AhR pathway through direct binding to the AhR-ARNT heterodimer and has antioxidant properties probably due to its chemical structure that may also derive from Nrf2 pathway activation [79]. It has been proposed that AhR/Nrf2 dual activation drives the efficacy of coal tar, a traditional topical treatment for psoriasis and AD that contains complex mixtures of polyaromatic hydrocarbons [67]. Furthermore, it has been showed that tapinarof induces mRNA expression of the late differentiation biomarkers, including FLG and IVL, leads to significant reduction of Th-17 cytokines, and reduces skin inflammation in an imiquimod treated mice [79].

In a phase 2b, double-blind, vehicle-controlled, randomized study, adolescent and adult patients tapinarof 1% cream demonstrated to be significantly more efficacious than placebo and tapinarof 0.5% cream in achieving primary endpoint (proportion of patients with an investigator global assessment (IGA) score of clear (0) or almost clear (1) or a ≥2-grade improvement in IGA score from baseline to week 12) [77]. This improvement was maintained for 4 weeks after the end of the study treatment [77]. The adverse reactions were more frequent for tapinarof compared to placebo, but they were mild to moderate in intensity [78].

## 6. Psoriasis

Psoriasis is a common, chronic, immune-mediated skin disease characterized by hyperproliferation of keratinocytes with consequent scaly, erythematous, and well demarcated plaques that can be painful and itchy [78]. Its pathogenesis can be explained by dysregulation of immunological cell function as well as keratinocyte proliferation/differentiation [78]. Although Th-1 over-activation was thought to induce psoriasis occurrence, it has been demonstrated that Th17 cells play a key role in psoriasis pathogenesis [78]. Th17 development is maintained by IL-23 mainly produced by dendritic cells. Th17 cells produce various cytokines, including IL-17A, IL-17F, and IL-22. IL-17A and IL-22 induce both keratinocyte proliferation, and tumor necrosis factor (TNF)-α, chemokine (C-X-C motif) ligand (CXCL)1 and CXCL8 production. TNF-α accelerates the infiltration of inflammatory cells, including lymphocytes, monocytes and neutrophils, from the peripheral blood into skin with dendritic cell activation.

In recent years, the involvement of AhR in the pathogenesis of psoriasis has been reported [80,81,82]. In murine and human models of psoriasis induced by imiquomod, the AhR stimulation with agonist FICZ resulted in attenuated psoriasiform skin inflammation, with milder parakeratosis and cell infiltration, a significant reduction in epidermal and scale thickness, and reduced expression of proinflammatory mediators [82]. Conversely, the blocking of AhR signal with the antagonist CH-223191 exacerbates psoriasis gene expression in patient biopsies [82]. A dysregulation of genes implicated in the catabolism of tryptophan has been described in psoriatic skin, leading to a low expression of naturally derived products, such as FICZ, and subsequently to a decreased activation of AhR [83]. In addition, in mouse models the AhR gene silencing exacerbates skin inflammation with upregulated gene expression of *IL-22*, *IL-17a,* and *IL-23* [82]. Indeed, the AhR signaling controls the expression of IL-22 and plays a central role in Th17 cells differentiation in vivo and in vitro [84]. Studies in psoriatic patients have shown an increased AhR expression in both peripheral blood mononuclear cells and skin biopsy samples [85,86]. These increased levels are correlated to increased levels of Th22 cells and IL-22 [85,86,87]. IL-22 inhibits terminal differentiation of keratinocytes and synergizes with the other pro-inflammatory cytokines inducing psoriasis-like epidermis alterations [84]. Recently, Cardinali et al. have tested the effects of two new synthetic AhR agonists, NPD-0614-13 and NPD-0614-24, in human epidermal and full-thickness reconstituted skin models of psoriasis [88]. These agonists are related to the natural agonist FICZ [67], have a pro-differentiating activity, and reduce the expression of pro-inflammatory cytokines and antimicrobial peptides [88]. Finally, emerging evidence in psoriasis indicated that vascular endothelial cells (VECs) participate in physiological and immunological functions such as in regulating leukocyte recruitment [89]. Recently, it has been demonstrated that the stimulation of AhR expressed on VECs is involved in the reduced neutrophil recruitment to the site of inflammation in psoriatic skin [89].

AhR stimulation is proposed as a therapeutic mechanism for the treatment of psoriasis, and tapinarof is one of the most studied topical drugs [90,91,92,93]. Its efficacy has been attributed to its capacity to modulate gene expression that leads to significant reduction of Th-17 cytokines implicated in psoriasis, including IL-17A and IL-17F, to increase antioxidant response and to regulate the skin barrier protein expression, including FLG and LOR [90,91,92,93]. In a phase 2b, double-blind, vehicle-controlled study in adults with psoriasis treated with tapinarof 1% cream, a statistically significant clinical improvement was demonstrated starting at week 2, maintained through week 16 [93]. The safety and effectiveness of tapinarof 1% cream once daily has been evaluated in two randomized phase 3 clinical trials completed in 2020 and awaits full publication (NCT02564042 and NCT03983980) [92].

## 7. Acne

Acne is a multifactorial inflammatory disease affecting pilosebaceous follicles [94]. Key elements in its pathogenesis are *Propionibacterium acnes*, keratinocyte hyperproliferation in the follicle, androgen-mediated increase in sebum production, and inflammation [94]. In recent years, several studies have been performed linking the expression of AhR and the appearance of acne [94,95]. In 2014, Fabbrocini et al. demonstrated the presence of an increased AhR expression in the skin lesions of patients affected by acne living in Campania (Italy), where epidemiological studies have suggested a possibly increased exposure to environmental dioxins [95]. The activation of AhR signaling pathway seems to have inhibitory effects in human sebocytes, with a reduction in sebum production [96]. An in vitro study conducted on immortalized sebocyte lines investigated the function of AhR in the control of sebum production and showed that its stimulation leads to an attenuation of the expression of genes involved in lipogenesis [97]. Probably, a cross-talk between AhR and Toll-like receptor (TLR)-2 is responsible for this effect [98]. On cultured human sebocytes the TLR-2 stimulation with the agonist peptidoglycan (PGN) induced secretion of inflammatory factors TNF-α and IL-8, but it is suppressed after knockdown of AhR and pre-treatment with the AhR antagonist CH223191 [98]. In addition, the AhR agonist TCDD enhanced TNF-α and IL-8 secretion in PGN-pretreated sebocytes [98]. Finally, Cao K. et al. have demonstrated that formalin-killed Corynebacterium acnes activates the AhR pathway in vitro, leading to inhibition of lipogenesis and induction of sebocyte differentiation [99]. Chloracne is an acne-like eruption subsequent to the exposure to high concentrations of polycyclic and halogenated aromatic hydrocarbons [100]. It seems to be associated with acceleration of terminal differentiation of sebocytes that highly express AhR [101]. Ligation of AhR by hydrocarbons stimulate the differentiation of sebocytes towards keratinocytes with secondary hyperkeratinization of pilo-sebaceum unit, resulting in the disease [101]. Cinnamaldehyde that inhibitsAhR-CYP1A1 signaling in sebocytes has been reported to improve chloracne [102].

## 8. Hidradenitis Suppurativa

Hidradenitis suppurativa (HS) is a chronic inflammatory disease of the hair follicle, affecting skin areas with apocrine glands [103]. It is characterized by the appearance of abscesses, fistulas, and suppurative cysts primarily in the axillary, inguinal, and anogenital regions [103]. Guenin-Macé L et al. have demonstrated that in HS patients an alteration in tryptophan catabolism induced by a normal bacterial skin flora, causes alteration in the production of AhR agonists [104]. These data suggest the hypothesis that the immune dysregulation underlying HS skin lesions may be caused by an alteration in the AhR pathway [105]. Furthermore, AhR activation has been shown to modulate the release of IL-17 by Th-17 lymphocytes, a cytokine that appears to be an important mediator in the pathogenesis of HS [105,106]. However certainly further studies are needed.

## 9. Conclusions

In recent years, an increasing number of studies have accumulated highlighting the regulatory role of AhR on the physiology of the skin. Consequently, its role in skin diseases has been researched. However, there is evidence of both beneficial and harmful effects of AHR signaling. Therefore, understanding of AHR function in the respective disease driving pathways is required. At present, most of the evidence concerns inflammatory skin diseases, in particular atopic dermatitis, psoriasis, acne, and hidradenitis suppurativa. Indeed, AhR altered function seems to be associated with both skin barrier impairment and releasing of proinflammatory cytokines, two of the pivotal factors of most chronic inflammatory diseases. These observations show that drugs acting on AhR could be useful in the treatment of such diseases. At the present time, the AhR agonist tapinarof, has shown, in a clinical trial, to be effective in the treatment of some of these diseases. Of course, more experience will be needed to confirm these data. Basic and pharmaceutical research will also be needed to better clarify the role of AhR in the physiological and pathological mechanisms of the skin, also with the aim of identifying new molecules targeting AhR for the treatment of skin, inflammatory, and other diseases.

## Figures and Tables

**Table 1:** Effects of activation of AhR signaling in inflammatory chronic skin diseases.

	Atopic Dermatitis	Psoriasis	Acne	Hidradenitis Suppurativa
**Effects of AhR Activation in Inflammatory Skin Disease**	Expression of genes encoding FLG, LOR, IVL, and other barrier-related proteins in the EDC loci Up-regulation of OVOL1 transcription factor important for the epidermal differentiation AHR axis activation inhibits the IL-13/IL-4-mediated STAT6 phosphorylation and restores the IL-13/IL-4-mediated FLG decrease Induction of ARTN gene expression	Significant reduction in epidermal and scale thickness with milder parakeratosis and cell infiltration Reduced expression of proinflammatory cytokines IL-22, IL-17a, and IL23 Reduced neutrophil recruitment	Inhibitory effects in human sebocytes, with a reduction in sebum production	Modulation in release of IL-17

Aryl hydrocarbon receptor (AhR); Filaggrin (FLG); Loricrin (LOR); Involucrin (IVL); Epidermal Differentiation Complex (EDC); OVO like1 transcription factor (OVOL1); Interleukin (IL); Signal Transducer, and Activator of Transcription 6 (STAT6); Artemin (ARTN).

## Data Availability

The datasets generated during and/or analyzed during the current study are available from the corresponding author on reasonable request.
